# Meeting Highlights: Genome Sequencing and Biology 2001

**DOI:** 10.1002/cfg.97

**Published:** 2001-08

**Authors:** Jo Wixon

**Affiliations:** Bioinformatics Division HGMP-RC Hinxton, Cambridge CB10 1SB UK

## Abstract

We bring you a report from the CSHL Genome Sequencing and Biology Meeting, which has a long and prestigious history. This year there were sessions on large-scale sequencing and analysis, polymorphisms (covering discovery and technologies and mapping and analysis), comparative genomics of mammalian and model organism genomes, functional genomics and bioinformatics.


**Comparative and Functional Genomics**
*Comp Funct Genom* 2001; **2**: 243–251.
**DOI:** 10.1002/cfg.97

**Meeting Highlights**

### Genome Sequencing and Biology 2001

### Cold Spring Harbor Laboratories, Long Island, New York – May 9th–13th 2001

Jo Wixon*, Managing Editor
*Bioinformatics Division, HGMP-RC, Hinxton, Cambridge CB10 1SA, UK*

*Correspondence to:
J. Wixon, Bioinformatics Division, HGMP-RC, Hinxton, Cambridge CB10 1SA, UK.

### Abstract

**We bring you a report from the CSHL Genome Sequencing and Biology Meeting, which has a long and prestigious history. This year there were sessions on large-scale sequencing and analysis, polymorphisms (covering discovery and technologies and mapping and analysis), comparative genomics of mammalian and model organism genomes, functional genomics and bioinformatics. Copyright © 2001 John Wiley & Sons, Ltd.**

### Large-scale sequencing and analysis

**Eric Green** started the meeting (by popular request) with a presentation charting the history of our progress towards the sequence of the human genome. This started with 'Early Heroes' featuring pictures of those who laid the foundations for the project, such as Mendel, Watson and Crick, Maxam and Gilbert, Sanger and Hood. Then he passed onto seminal papers in the field of genomics, showing the covers of 'Genome Issues' of Science and other journals and magazines, featuring the genome sequences of model organisms completed prior to the human genome project. Then, set to the 'Mission Impossible' theme tune, he highlighted those people who have tirelessly campaigned for funding for the Human Genome project, followed by a celebration of the achievements so far, culminating with the release of the draft sequence.

**Lisa Stubbs** (**US Department Of Energy – Joint Genome Institute**) reported on a comparative analysis of human chromosome 19 and the related regions of the mouse genome (parts of chromosomes 7, 8, 9 and 10). The comparison has confirmed 128 predicted human genes which previously only had EST evidence and has increased the gene count of human chromosome 19 to ~1,200. 80% of human exons were conserved in mouse. Whilst they found that unique human genes were almost always conserved, the locations of gene clusters are conserved, but the numbers of each type of gene, and their organisation are different.

**Rick Wilson** (**Washington University, St Louis, Missouri**) presented observations on the sequence of the non-recombining region of chromosome Y (NRY). This chromosome has a large region of heterochromatin on its q arm and around 3 Mb of sequence on its tips that recombines with the X chromosome. They have currently sequenced ~8 Mb on the p arm and ~15 Mb on the q arm. In general the sequence is extremely repetitive and there are few single copy markers. The gene density is low, at 6.4 genes per Mb it is around half that of the average across the genome. Deletions of parts of Y are known to lead to male infertility, one such region lies next to the end of the p arm. This was found to be a repeat region that includes a 3.5 Mb palindrome of four replicons. The group think that homologous recombination between the ends of this region cause the deletions.

**Ian Dunham** (**The Sanger Centre, UK**) presented a report on progress with the sequence of chromosome 22 since publication, which went ahead with 12 gaps. Some gaps have been closed using bridging clones and mouse clones, but in some cases, even the bridging clones were shown by fibre-FISH to have deletions. Three such gaps remain, which they describe as 'uncloneable' regions. There seems to be a correlation between high GC content and difficulty in cloning gaps. In their first round annotation, 72 Unigene clusters only had 3′ exons detected in the draft, and there were many partial genes. After attempts to resolve these problems and the inclusion of new expression data, the number of

Copyright © 2001 John Wiley & Sons, Ltd.

known genes has risen from 247 to 368, 49 genes have been fused to make 23 genes and the number of pseudogenes has gone up from 134 to 162. Their readjusted estimate for the number of human genes (based on this data and that for chromosome 21) is 32,000.

**Nobuyoshi Shimizu (Keio University, Japan)** gave a report on progress with chromosome 21. Of the 225 genes they predicted in their initial publication, 127 were known and 98 were predicted, now they have 153 known genes and 99 predicted ones, taking the total to 252. There were three gaps totaling 100kb when the sequence was published, one of these has been closed using bridging clones and PCR, reducing the total gap length to 50 kb.

**Richard Mural (Celera, Maryland)** spoke about their shotgun assembly of the mouse genome. Sequencing to 5.5X coverage of DNA from four mouse strains has taken them about a year. They have 86% of the genome as scaffolds over 1 Mb long, and about half the genome is in scaffolds over 5 Mb in length. Their preliminary annotation of the mouse genome sequence indicates that it has a similar gene to count to the human genome. They have found 2.7 million candidate SNPs, but admit that some of these could be due to sequencing errors. Within strains they see $\sim 1$ variation per $10^4$ kb, between strains the level of variation is higher, with one potential SNP per 2 kb.

**Steve Scherer (University of Toronto, Canada)** spoke about the genes that have been identified from the sequencing of human chromosome 7. They predict that there are 2147 genes, 762 of which are full length and have been characterised. There are several genes over 500 kb long on the chromosome, and one is 2.3 Mb long (FISH probes for the ends of this gene can be seen as discrete spots on chromosomes). 39% of the genes appear to be alternatively spliced. They have identified a duplicated sequence flanking a region commonly lost in Williams-Beuren syndromes. They are now looking at tissue distribution of expression and imprinting of the predicted genes.

**Evan Eichler (Case Western, Cleveland)** presented the results of a study of recent duplication events on chromosome 16p. The group has identified a 20 kb low copy repeat region present in 15 copies, some of which are in tandem. Comparative analyses have shown that this expansion occurred at the time when the human and great ape lineages separated, and that there is higher conservation of introns than exons in the *morpheus* gene family found in the region when compared to mouse, indicating positive selection acting upon these genes. This could indicate that there will be recently emerged, rapidly evolving genes in our genome that will not have definitive orthologues in model organisms.

**Jim Kent (University of California, Santa Cruz)** talked about recent refinements to their Human Genome Browser (**http://genome.ucsc.edu**). The browser displays the 'Golden Path' genome assembly with the chromosome at the top and beneath that, 25 'tracks' of other data, compiled with the help of collaborating groups. Known genes, for example, are marked in blue, with boxes for exons and lines for introns, and arrows indicating the direction of transcription. Other tracks include ESTs, SNPs, genetic markers and pufferfish homologies. The group have added a new search tool, BLAT, which is like BLAST, but faster, users can paste in their sequence and search against the draft. They are working on adding the data from the mouse genome effort, currently at around 3X coverage. They hope this will highlight conserved exons and regulatory regions.

**David Lipman** of the **NCBI** spoke about their genome assembly **http://www.ncbi.nlm.nih.gov/cgi-bin/Entrez/hum_srch**. They hope to shift to making a new 'build' every two weeks, to cope with the flow of sequence updates and cDNA data. They start with a sequence layout, which includes multiple types of data, from STS data to personal communications, and then make the sequence build. This involves melding of overlaps, ordering and orienting of contigs and finally the build is compared to the mouse data to confirm the veracity of the assembly.

### Polymorphisms I: discovery and technology

**James Mullikin (Sanger Centre, UK)** spoke on behalf of The International SNP Map Working Group. They have made a map of 1.42 million SNPs, from The SNP Consortium and overlap analysis by the Human Genome Sequencing Consortium, across the genome. On average, there is one SNP per 1.9 kb, but the density varies across the genome 8-fold from the mean. They estimate that 60 000 of the SNPs are found in exons and that as many as 93% of gene loci have one or more identified SNPs.

**Gabor Marth (NCBI, Maryland)** presented the

Copyright © 2001 John Wiley & Sons, Ltd.

results of a study using SNPs found in overlapping sequence data to look at population history. Using a strict criterion of 99.75% similarity they identified overlaps representing ~23% of the genome in which they searched for SNPs. The correlation between their genetic diversity data and the established model of a small historical human population followed by population explosions, was very poor, in fact the data better fits a model of a two-stage population history with a bottleneck period. Their model also predicts much greater linkage disequilibrium (LD) than was previously thought.

The remaining talks in this session described SNP detection strategies or genotyping approaches. **Sanjay Tyagi** (**Public Health Research Institute, New York**) talked about a molecular beacon approach to genotyping. This uses fluorescently labeled, allele specific hairpin probes in which different fluorophores are held close to a quencher. Whichever probe binds to its target (say, the 'wild-type' allele) can then emit light, and so detection of both wavelengths would indicate a heterozygote.

**Mostafa Ronaghi** described the pyrosequencing strategy of **ParAllele Genomics** (**Palo Alto, California**). In this 'sequencing by synthesis' system, the ATP released when the correct nucleotide is provided for the synthesis reaction causes luciferase to emit light. This system has been applied both to detection and genotyping.

**Patrick Cahill** of **Genome Therapeutics Corp**. (**Waltham, Massachussetts**) presented their Exo-proofreading SNP assay for genotyping. The set-up uses two fluorescent dyes, one of which is activated upon detection of the 'wild-type' allele and one upon detection of the 'mutant', with both being activated by a heterozygote sample.

**M. Olivier** (**Stanford University, California**) presented an improvement upon the Invader system of Third Wave Technologies. They spot genomic DNA from multiple samples onto glass slides, perform the reaction (to detect the same SNP in each sample) using much smaller volumes of reagents than before and then detect the fluorescent signals generated using a standard microarray scanner.

**David Cutler** (**Johns Hopkins University, Maryland**) presented a method for SNP discovery that uses Affymetrix chips. The chips are loaded with four rows of oligos representing the sequence of a chosen region of the genome; in each column the rows have each of the four bases in one position. The chip therefore makes a call for every base, effectively resequencing the region, discovering and genotyping SNPs at the same time.

**Mark Chee** (**Illumina Inc, California**) described a bead-based array system which can be combined with multiplexed genotyping assays (based on oligonucleotide ligation and PCR amplification) in a matrix designed to match the 96-well plate format for high-throughput.

### Polymorphisms II: mapping and analysis

For this session we report just those talks or parts of talks of more general interest, covering new approaches or practical observations across the entire genome, rather than theoretical discussions relating to genetic analysis approaches.

**Päivi Onkamo** (**Universty of Helsinki, Finland**) described a data-mining based method (Haplotype Pattern Mining) to find combinations of variations that are associated with a trait. The system involves scoring the haplotypes, and building a table of recurrent haplotypes in order of association score with the disease. The table is then cropped to remove those below a minimum association cut-off. The remaining markers are then scored for how many times the most common allele segregates with the disease, which narrows down the region containing the true disease predisposing mutations and may be able to spot multiple loci.

The remaining talks in this session were concerned with linkage disequilibrium (LD) studies. LD denotes the occurrence of strings of alleles occurring together in a population more frequently than would be expected, because the sections of chromosomes on which they reside descend from the one ancestral chromosome.

**David Reich** (**Whitehead Institute, Massachusetts**) presented a systematic assessment of LD in the human genome. The group has performed SNP discovery and genotyping in 19 regions of the human genome in 44 Utah samples, generating sequence from patches set at known distances apart, to allow them to assess how far LD typically extends. Their results indicate that LD extends around 60 kb in either direction from common alleles, which is much more than was expected. The results for 48 Swedish samples were near identical. In Nigerian samples LD was much lower and dropped off much sooner, but any long stretches were always conserved in the European origin samples. This difference must be due to a different

Copyright © 2001 John Wiley & Sons, Ltd. 

population history and they found that their data supports a severe bottleneck event in the European population ~27–53 000 years ago, which could represent the original migration 'out of Africa'.

The **Sanger Centre** and the **Wellcome Trust Centre for Human Genetics** have collaborated on an LD map of human chromosome 22. **Elisabeth Dawson** (**Sanger Centre, UK**) presented their results. They have genotyped SNPs at 7-15kb intervals across the long arm of the chromosome in seven CEPH families and are now working on samples from 92 unrelated individuals. To date they have data from 1358 markers, ~1000 of which are less than 30 kb apart and cover 14 Mb of the chromosome. 75% of marker pairs that are 10 kb apart demonstrate detectable LD, this falls to 65% of pairs at 10–20 kb spacing, and just less than 50% of pairs at 30 kb spacing and over. Using a cut-off LD score of 3.3, they estimate that LD extends around just 30 kb on average, although chromosome 22 is thought to have higher recombination than other autosomes, which could explain this lower figure. They see a fairly good correlation between LD and recombination frequency (genes not separated by recombination have high LD) and see a strong association with gene density.

An LD study comparing multiple regions of the X chromosome with autosomal regions was reported by **Michael Zwick** (**Johns Hopkins University, Maryland**). They looked at 8 X-linked regions and 32 autosomal regions in 40 samples of mixed ethnicity from the NIH Polymorphism Discovery Resource. They have seen a large variation in the distance over which LD extends, from 2 kb to 100 kb, with an average of 40–70 kb. Chromosome X has more regions showing significant LD, and LD extends further on X than on autosomes. They see an excess of rare sites both on X and autosomes and he commented that SNPs making changes in proteins were the most rare, which he thinks will make association studies difficult.

**Mark Daly** (**Whitehead Institute, Massachusetts**) presented the results of a fine structure LD map across 400 kb on 5q31, in which they discovered and genotyped ~100 SNPs. A heterozygosity plot of their data showed two troughs (haplotypes) split by a peak. On further investigation they found that each trough region had two main haplotypes, one at around 75% frequency and one at around 20% frequency, the pairs with similar frequencies being linked, but split by one region showing 8% exchange, explaining the peak between the troughs. If these observations can be extended to the whole genome, they feel that the prospects for LD mapping are good.

### Comparative genomics I: model organisms

The **Salk Institute/Stanford/Plant Gene Expression Center (PGEC) consortium** is identifying full-length *Arabidopsis* ORFs to enable them to produce an Affymetrix chip for *Arabidopsis*. **Athanasios Theologis** (**PGEC, California**) described how they use a chip loaded with oligos representing the genomic sequence and hybridise mRNA from *Arabidopsis* plants exposed to different conditions to help verify the genome annotation. Of the 783 genes they have analysed so far, 221 have a different structure than was predicted. Another part of the project is to construct the 'ORFeome' of *Arabidopsis*, which means full-length clones of the ORFs of all genes.

The **US Department of Energy Joint Genome Institute** sequenced 15 microbial genomes to ~8X coverage in October 2000. **Paul Predki** talked about the observations that the team has made, now that they have produced 18 completed or 8X draft microbial genomes. They have found that at 3X it is possible to find some part of all genes, whilst at 7.5X, 95% of genes have high quality data. They use the Rolling Circle Amplification kit (Pharmacia) to amplify microbial and mitochondrial genomes and vectors straight from the cells.

**Paul Cliften** (**Washington University, St Louis, Missouri**) spoke about a project to identify regulatory regions in the yeast *Saccharomyces cerevisiae* by comparative genomics. They have sequenced between 1000 and 2000 random genomic clones (and the promoter regions of several chosen genes) from a selection of yeasts. These included species such as *S. paradoxus, cariocanus, mikatae* and *bayanus*, which showed high homology in coding regions and some homology in non-coding regions and species such as *S. castellii, kluyveri, unisporus, dairenensis* and *exiguus*, which showed much lower homology, especially in non-coding regions.

The Génolevures project identified over 20 000 novel genes in 13 different yeast species. **Bernard Dujon** (**Pasteur Institute, France**) presented their results, and explained that the data can be retrieved from **http://cbi.labri.u-bordeaux.fr/Genolevures/**. The project uncovered a significant number of 'Ascomycete-specific' genes and was also used to revisit

Copyright © 2001 John Wiley & Sons, Ltd. 

the annotation of *S. cerevisiae*. In collaboration with Génopole (at the Pasteur Institute) *Candida glabrata* has recently been added to the study, sequencing to 0.5X coverage has resulted in the discovery of 2000 novel genes from this yeast.

**Steve Johnson (Washington University, St Louis, Missouri)** talked about zebrafish genomics, in particular describing a project to generate expressed sequence tags (ESTs) and place them on the zebrafish genomic map. Of 85 000 ESTs, they have so far mapped 8000 and analysed 66 000, identifying 17 500 genes, 4150 of which have human homologues. Based on the percentage of known genes they have detected, they predict that they have 60% coverage of an estimated 29 000 genes. The mapped clones have uncovered several good stretches of synteny with the human genome, however, it seems quite common to find two zebrafish genes per human homolog.

**Andrew Fraser (Wellcome/CRC Institute of Cancer Research and Developmental Biology, UK)** described how a recent RNA interference (RNAi) project has provided phenotype data for just less than 90% of the genes on *C. elegans* chromosome I. As a result of this work, the group has assigned function to 13.9% of the genes analysed, increasing the number of genes with known phenotypes on that chromosome from 70 to 378. The group has now extended their study to include chromosomes II and X and have ~41% of the genome cloned. About 12% of genes show a detectable phenotype and they have an ~80% success rate for detection of known embryonic lethals.

Since the *Drosophila* genome was published in March last year with 1630 gaps, **Susan Celniker (Lawrence Berkeley National Lab, California)** and colleagues have been busy bringing that release up to fully finished standards, and have closed 330 gaps so far.

They are using EST data to look beyond the initial gene estimate, and have discovered more genes, but won't yet say how many. Other projects include insertional mutagenesis (they hope to target 70% of genes), gene expression pattern profiling of 700 transcription factors using RNA *in situ* hybridisation, and comparative genomics (they are sequencing *Drosophila pseudoobscura*) to help the search for regulatory regions. Their current 'promoter bashing' approach involves searching for known and overrepresented sequences and plotting these onto the sequence, removing singletons and homing in on clusters often yields genuine hits on promoters.

**Marc Vidal (Harvard Medical School, Massachusetts)** presented the *C. elegans* ORFeome project. Predicted ORFs are PCR amplified from a cDNA library using ORF-specific primers and cloned using a recombination cloning system. The clones are sequenced to generate ORF sequence tags that are used to verify identity and splicing. At least 70% of almost 10,000 genes that were predicted *ab initio* have been verified in this way, however, 27% of these experimentally confirmed genes show a different structure than that predicted by GeneFinder. They have evidence that supports the existence of 17,300 *C. elegans* genes and their clones will provide a resource for an array of functional genomics approaches.

### Comparative genomics II: mammalian genomics

For this session we provide coverage of those talks detailing resources, new approaches or observations made across whole genomes, rather than detailed studies of single genes or gene families.

**Robert Strausberg** of the **National Cancer Institute** spoke about their Mammalian Gene Collection (**http://mgc.nci.nih.gov**), a set of full length cDNA clones whose sequences are deposited in GenBank. Whilst he admitted that RefSeq currently has more, longer cDNAs, they are planning to switch MGC to using longer clones. They estimate that they currently have full length clones for ~42% of the human genome.

Primate genomes are much too similar to our own to be of any use in searching for conserved regions, rather they have value in pinpointing differences, as **Ines Hellman (Max Planck Institute for Evolutionary Anthropology, Germany)** explained. They have sequenced 10,000 clones from a random shotgun library to obtain 0.1% of the chimpanzee genome. On average they see only 1.26% difference across the genome, the X and Y chromosomes are the main outliers, with X showing the least differences and Y showing the most. They are also comparing ESTs (to look at changes in coding sequence) and transcriptome data (to look at expression pattern differences) across a range of primate species.

**Gabriela Loots (Lawrence Berkeley National Laboratory, California)** presented an approach to identifying trancription factor binding sites which combines a search for sites using Transfac, with sequence alignments between human and mouse.

Copyright © 2001 John Wiley & Sons, Ltd.

They used a region on human 5q31 as a test, looking for GATA3 promoters in this cytokine gene rich region. Whereas the Transfac search predicted GATA3 sites randomly across the promoter regions of all 18 genes in the region, the comparative sequence analysis confirmed only those in the promoter regions of the cytokine genes, which have been characterized as GATA3 responsive.

A comparative sequence analysis of large regions in 12 vertebrate species was described by **Jeff Touchman** (**NIH-Intramural Sequencing Center**). Based on the availability of BAC libraries they chose chimp, baboon, pig, cow, cat, dog, mouse, rat, chicken, zebrafish and *Fugu*. The first part of the study is comparative mapping against human of four initial targets covering some 10 Mb. Using human mouse comparisons, they identify highly conserved regions and use these as probes to pull out the corresponding clones from the BAC libraries. All clones are sequenced to 8–10X, and target one, the CFTR region, is now almost complete. Chimp and baboon give the best matches as expected, followed by cat, dog, cow and pig, which group together. Mouse and rat give poorer homology scores than would be expected compared to the results of the other species, considering their evolutionary position. They find three way comparisons give much more solid results, such as mouse and rat to human or *Fugu* and zebrafish to human.

An approach using mouse whole genome shotgun data to help predict human genes was described by **Roderic Guigo** (**Genome Informatics Research Lab, Spain**). The method uses tBLASTx to make a rapid comparison between the human and mouse sequences and combines these results with geneid (an *ab initio* gene prediction program). Both programs score probable coding regions using likelihood ratios, which can be combined. Unlike previous methods, this can work with fragmentary data such as shotgun data, and from tests with simulated data, they estimate that 3X data will be sufficient to effectively predict human genes. Using the current mouse coverage against the October 2000 freeze of the Golden Path, they found 4,050 new genes, which they clustered into families, resulting mainly in ~3,800 singletons and several hundred 2 member families. This could mean that there are several 1,000s (rather than tens of 1,000s) of genes still to be found.

**Douglas Mortlock** (**Stanford University, Califonia**) reported on an approach to identify cis-acting regulatory elements by using transgenic BAC scanning and comparative analysis. It is quite common for mammalian genes to be regulated by cis-acting elements at some distance from the genes themselves. This approach uses homologous recombination in bacteria to generate a series of truncated BACs. If the gene of interest is lacZ tagged, the effects of the truncations can be assessed by making transgenics, which then identifies regions critical for expression of the gene. Once these have been identified, sequencing and comparison of the region in human and mouse is used to identify conserved sequence elements within the critical regions. This approach has already been used to identify sequences involved in regulation of joint patterning during skeletal development.

### Functional genomics

High-density oligonucleotide arrays can be used to detect tracts of conserved elements between two genomes, **Kelly Frazer** (**Perlegen, California**) described the use of a human chromosome 21q array to detect conserved elements in the genomes of mouse and dog. Typically, the mouse probes show that there are short conserved regions of 30–60 bp, these are then grouped with any neighbours less than 100 bp away (this occurs more commonly in exons). Many of the shorter, commonly non-exon, elements were not conserved in dog, whereas the larger elements were detected in both comparisons. Only about half of the longer elements represent known genes, some have no match in other sequence databases and there are some conserved non-exon elements.

The ***Saccharomyces cerevisiae*** **Genome Deletion Consortium** has produced deletions of every yeast gene, each one coded with a unique tag. **Adam Deutschbauer** (**Stanford University, California**) described a project that used 4657 of these strains to identify genes that are important for sporulation and germination. The group has identified ~200 genes required for sporulation (including the majority of previously known sporulation mutants) and ~50 genes required for germination. They also note that the majority of meiosis specific genes are not required for efficient sporulation.

**Derek Symula** (**Wadsworth Center, New York**) described a high-throughput strategy for isolating mouse mutants. The strategy involves making mutant embryonic stem (ES) cells *in vitro* (which

Copyright © 2001 John Wiley & Sons, Ltd. 

can be stored), screening them using microarrays to identify interesting mutants and selecting these for production of mutant mice. They have tested their idea using a pair of heterozygous mutant strains carrying overlapping deletions; as expected they see down regulation in both strains of genes in the overlap region and in only one strain in the other regions. Tissues from mice produced using these cell lines showed expression patterns similar to those of the ES cells and observations from the ES cell data did appear to relate to the phenotypes observed.

The RIKEN mouse full-length cDNA collection and its functional annotation have generated much international interest; **Yasushi Okazaki (RIKEN, Japan)** presented a report on their progress. Over 20 000 clones (of average size 1.2 kb) have been sequenced, yielding 15 000 clusters of cDNAs. The Functional Annotation Of Mouse (FANTOM) meeting in September 2000 led to the development of FANTOM+, a web-based system for annotating the clones. This incorporates Gene Ontology consortium (GO) and TIGR EGAD functional classifications, tissue expression patterns, sub-cellular localization and mutation data.

**Matthew Meyerson (Harvard Medical School, Massachusetts)** presented a novel method for the discovery of infectious agents. There are several human disorders, including some inflammatory diseases and cancers, that are thought to be related to infectious agents, but for which no microbe has been identified. Microbes can be hard to culture, so existing methods are commonly PCR-based, to avoid this step. This approach however, uses computational subtraction, relying on the fact that mRNA extracted from infected tissue will also contain genomic DNA of infectious agents. Taking the 'Refseq' mRNA collection, a MEGABLAST search was used to remove human and mouse genome matches, human mitochondrial sequences and repeats, and vectors. Poor quality sequences were also removed, leaving 2% of the ESTs. Amongst these the group found matches to *Pseudomonas aeruginosa, Hepatitis, Helicobacter pylori, Salmonella* (displaying the expected tissue specificities) and cytomegalovirus and papillomavirus.

**Yutaka Suzuki (University of Tokyo, Japan)** described a project to identify the potential promoter regions (PPRs) of 1031 known human genes and search within those for transcriptional start sites. Using mRNAs from cDNA libraries prepared by the 'Oligo-capping' method, to get the full 5′UTRs, the group aligned these mRNAs with genomic sequence and retrieved adjacent sequences as their PPRs. These regions were then screened for known promoter elements; 97% had a GC box, 64% had a CAAT box, 32% had TATA boxes and 85% had initiators. About half were located in CpG islands, with TATA box containing PPRs less likely to be in CpG islands. The group also noted that for each gene, the position of the mRNA start site can vary, over a range of about 60 bp.

**The German Human Genome Project** has a network to identify and sequence novel human genes, and functionally characterize the encoded proteins, **Stefan Wiemann (German Cancer Research Centre, Germany)** reported on their progress. During the last three years, over 50 000 ESTs have been completely sequenced, generating a set of full-length cDNA clones representing ~2400 genes, over 800 of which are novel (**http://www.dkfz-heidelberg.de/abt0840/GCC**). These ORFs are then cloned into N and C-terminal GFP fusion constructs for subcellular localisation experiments (this allows for the potential of the tag to affect localization, since the two results should match). The team is making video footage to observe any movements of the proteins, one example of what they have seen is shuttling between the golgi apparatus and the cell membrane.

**Timothy Aitman (Imperial College, UK)** presented a combined microarray gene expression profiling and genetic linkage analysis approach for looking at complex trait genes. They are using the spontaneously hypertensive rat (SHR) as a model of human hypertension. Using microarrays they identified 329 genes which were differentially expressed in the SHR rat and then looked for those which mapped to the quantitative trait loci that they identified by genetic linkage analysis. Of 18 such genes, three mapped to QTLs for dyslipidaemia and three to QTLs for blood glucose. One gene, *Cd36*, was shown to be knocked-out in the SHR due to a chromosomal duplication/deletion and underlies metabolic QTLs on chromosome 4.

### Bioinformatics

This session started with presentations on three genome assemblers, **Paul Havlak (Baylor College of Medicine, Texas)** started the session with a presentation of the Atlas assembler for mammalian genomes. Atlas combines clone-based and whole-genome shotgun data, and uses a hierarchical

Copyright © 2001 John Wiley & Sons, Ltd.

assembly strategy, with the aim of being able to assemble mammalian genomes in a matter of days. The core of the method is a sampling-based, high throughput sequence comparison tool, which identifies read overlaps and repeats (samples with excessive matches).

**Pavel Pevzner** (**University of California, San Diego**) talked about a new algorithm, called 'Euler'. This approaches assembly in a counter intuitive way, cutting up all the reads into smaller fragments, which pushes the problem to be solved from being a difficult Layout Problem to a Eulerian Path Problem with polynomial algorithms for DNA sequence assembly. This Eulerian Superpath approach offers new possibilities for repeat assembly and he claims that it resolves all repeats except long perfect repeats.

Arachne is another whole genome shotgun assembler, which was described by **Serafim Batzoglou (whitehead Institute Massachusetts)**. Arachne can rapidly and accurately assemble paired sequence reads, and looks at 24mers in reads, sorts them and searches for other reads containing those 24mers to build alignments. Very dense and overcollapsed contigs with inconsistent links are typically repeats and these are removed. It looks for contigs with multiple links to another contig and then fills in the gaps by putting the repeats back in, using the link data.

**Ewan Birney** (**European Bioinformatics Institute, UK**) gave a presentation on EnsEMBL, a joint human genome annotation project between The Sanger Centre and the EBI (**http://www.ensembl.org/**). He stressed the open culture of the resource, pointing out that the data and the source code of the software are freely available and that they are happy to collaborate with other groups on development. EnsEMBL maps $\sim$90% of human and $\sim$75% of rodent cDNAs. They use InterPro for first round functional annotation and TRIBE for gene clustering. The team are also applying the EnsEMBL analysis pipelines to the mouse sequence, and are matching mouse reads to the human genome.

**Ian Korf** and **Michael Brent** (**Washington University, St Louis, Missouri**) presented Twinscan (**http://genes.cs.wustl.edu/**), a new system for high-throughput gene structure prediction. Twinscan exploits patterns of conservation observed in local alignments between homologues, simultaneously modeling gene structure and evolutionary conservation. Twinscan can handle multiple or incomplete matches and allows for inversions, duplications and differences in exon-intron structure between the target sequence and its homologues. Currently Twinscan is optimised for human-mouse comparisons, but the group plans to adapt it for a wide range of genomes.

**Mark Yandell** described **Celera**'s automated approach to annotation of the human genome. The first stage is a suite of analyses including RepeatMasker, Genscan and BLAST, which marks up features. These results are passed onto an auto-annotation algorithm called Otto, which uses these to infer the locations and structures of genes. Each Otto annotation is then checked by BLAST, analysed using InterPro, assigned Gene Ontology terms and classified into a family. The 'trail' of evidence leading to the assignments is stored as an XML file and loaded into a relational database. Their current human gene count estimate (including their 14,000 genes with only 1 supporting piece of evidence) is $\sim$40,000.

Programs for identifying internal exons of genes are now highly sophisticated and accurate, however, over 40% of first exons are non-coding, and the remainder are partially coding, making them much harder to identify. **Ramana Davuluri** of the **Cold Spring Harbor Laboratories (New York)** described their attempts to identify 5′ terminal exons in the human genome. They have made a first exon database using data from a full-length cDNA sequencing project and have taken 90% to use as a training set and 10% to test their tool. First they try to identify the donor site GT, then they look for the maximum CpG% in a sliding window ($\sim$70% of first exons are CpG rich), then they look for potential promoter sites and try to assign the first exons. Whilst their tool is much better at finding CpG island first exons, they do have an $\sim$65% overall success rate.

Small RNAs are non-coding, functional ORFs such as tRNA, rRNA, snRNA and snoRNA. The existing tools only look for known small RNA types, whereas the tool described by **Elena Rivas** looks for novel ones. The program uses the facts that these genes exhibit base composition effects and have secondary structures. It assumes that mutations in these genes observed by comparing related species will show a pattern of compensatory base changes that would conserve the base-paired secondary structure. In alignments of E. coli with other bacteria they have predicted 130 novel small RNAs and of 50 they have tested so far, 12 have been confirmed.

Copyright © 2001 John Wiley & Sons, Ltd.

### Keynote speakers

**Eric Lander** spoke about variation in the human genome, commenting that it occurs at 1 base per 13 000, which appears to be very low. Looking at Crow's formula for heterozygosity in a population at equilibrium, this low diversity must reflect our small founding population, rather than the huge size our population has reached today. He is particularly interested in linkage disequilibrium (LD), and asked 'how many segments of LD are there?' He cited the work of Reich and colleages, and Bolk and colleagues (see Polymorphisms II: Mapping and Analysis), which show that LD extends roughly 60 kb in both directions in populations of European origin. They have seen that the fine structure of LD is in blocks, with only 2–4 common alleles. He suggested that these common variants may well underlie many common diseases. Older regions have shorter LD blocks, whereas younger regions have longer LD blocks. In conclusion, he recommended a switch to a haplotype map, in which ancestral blocks should be defined for large populations. This would then help to reduce the number of markers that need to be typed in genetic scans. He called for broad collaboration and an open, inclusive structure with public data releases, and a common sample collection, in which 300,000 SNPs would be typed.

**Paul Nurse** announced the almost completion of the *Schizosaccharomyces pombe* genome (one cosmid is still in progress). There are three chromosomes; I (5.7 MB), II (4.6 MB) and III (3.5 MB), and 12.5 MB of sequence from these has been deposited in the EMBL database. However, since the sequence has not yet been published, we are not in a position to bring you too many details of his talk, but we shall be featuring *Sz. pombe* in our next issue, which is out in October. The team predict ~5000 genes, which is less than some prokaryotes, here Paul asserted that being a eukaryote does not depend on how many genes you have, but what you do with them. Unlike *Saccharomyces cerevisiae*, the *Sz. pombe* genome does not show much evidence of duplication, the vast majority of its genes are unique. Around half of the genes have introns, and *Sz. pombe* has bigger intergenic regions, including extended upstream regions of genes, than *S. cerevisiae. Sz. pombe* has around 50 genes that are highly homologous to known human disease genes, the majority of these are cancer related and the next biggest grouping are for metabolic disorders, both of which suit the strengths of *Sz. pombe* as a model organism.

---

The Meeting Highlights of *Comparative and Functional Genomics* aim to present a commentary on the topical issues in genomics studies presented at a conference. The Meeting Highlights are invited and each represents a personal critical analysis of the current reports and aim at providing implications for future genomics studies.

Copyright © 2001 John Wiley & Sons, Ltd.

